# Navigating abstract virtual environment: an eeg study

**DOI:** 10.1007/s11571-016-9395-z

**Published:** 2016-07-20

**Authors:** Alireza Mahdizadeh Hakak, Joydeep Bhattacharya, Nimish Biloria, Roy de Kleijn, Fanak Shah-Mohammadi

**Affiliations:** 1 Faculty of Architecture, TU Delft, Delft, The Netherlands; 2 Department of Psychology, Goldsmiths University of London, London, UK; 3 Leiden University, Leiden, The Netherlands; 4 Azad Islamic University, Tehran, Iran

**Keywords:** EEG, Abstract environments, Fully designed, Semi-designed, Perception

## Abstract

Perceptions of different environments are different for different people. An abstract designed environment, with a degree of freedom from any visual reference in the physical world requests a completely different perception than a fully or semi-designed environment that has some correlation with the physical world. Maximal evidence on the manner in which the human brain is involved/operates in dealing with such novel perception comes from neuropsychology. Harnessing the tools and techniques involved in the domain of neuropsychology, the paper presents nee evidence on the role of pre-central gyrus in the perception of abstract spatial environments. In order to do so, the research team developed three different categories of designed environment with different characteristics: (1) Abstract environment, (2) Semi-designed environment, (3) Fully designed environment, as experimental sample environments. Perception of Fully-designed and semi-designed environments is almost the same, [maybe] since the brain can find a correlation between designed environments and already experienced physical world. In addition to this, the response to questionnaires accompanied with a list of buzzwords that have been provided after the experiments, also describe the characteristics of the chosen sample environments. Additionally, these results confirm the suitability of continuous electroencephalography (EEG) for studying Perception from the perspective of architectural environments.

## Introduction

Spatial navigation is a dynamic and intricate brain function required to locate oneself in space, which is vital for human’s survival in daily life. Integration of sensorimotor information is required for navigation: subject will associate external sensory stimuli with sensory commands. Individuals for instance receive external stimuli such as building and pathways and internally create mental representations of spatial maps and subsequently use this information to navigate in the environment (Brunsdon et al. 2007; Davis 1999; Farah 1989). Therefore, individuals are required to create a mental image of the environment which they are navigating and with respect to their target, they manipulate their current position (Palermo et al. 2008). This suggests that the neural computation to output motor command required for spatial navigation activates various cortical regions distributed over the brain. Recent noninvasive studies using virtual environments have highlighted the brain regions related to spatial information processing and navigation; the hippocampus, parahippocampal gyrus, posterior cingulate gyrus, temporal cortex, insula, superior and inferior parietal cortex, precuneus, dorsolateral prefrontal cortex, medial prefrontal cortex, premotor area and supplemental motor area are all activated during these tasks (Aguirre and D’Esposito 1997; Burgess et al. 2001; Hartley et al. 2003; Iseki et al. 2008; MacEvoy and Epstein 2007; Maguire et al. 1998; Spiers and Maguire 2007a, b, c; Wolbers et al. 2007). Simultaneous activation of many cortical regions inferred from navigation, should be integrated and functionally connected as coherent activity across different brain areas is important for cognition and action (Singer 1999; Varela et al. 2001).

This new-found knowledge about the understanding of brain network underlying spatial navigation acquired by the advent of modern neuroimaging techniques has greatly stimulated the field of Architecture (Eberhard 2008). For example, a typical question a [spatial] designer, namely an architect, has to consider even before starting the design process is how humans, i.e. the users of the designed environment, will perceive the environment. Given that a significant portion of our time is usually consumed in built environments, a better understanding of human brain’s responses to different designed environments would invariably improve the efficacy and intended purpose of the design. This is the primary motivation of our study in which we monitored large scale electrical activities of humans while they were virtually perceiving/navigating in three different designed environments, fully-designed, semi-designed and abstract design environment.

Architecture is a multi-faceted and multi-function discipline, which involves the act of visualizing, designing and problem solving as an iterative process. Studying the manner in which architects operate reveals the prevalence of a divergent approach during the phase of form finding as opposed to a convergent approach being employed during the problem-solving phase in order to narrow down appropriate design solutions and for subsequently finding the best one. The neural correlates of these two design phases, divergent and convergent, are different (see for example, (Limb and Braun 2008) on divergent/convergent thinking in the context of musical improvisation) and it would be of benefit to an architect to discover this difference in the brain’s functioning so that they can combine the respective potentials in the most appropriate and efficient manner. For example, it could be expected that exposure to an abstract environment at the early stages of design could help the designer suspending variety of potential solutions and therefore promoting divergent thinking (Ritter et al. 2012).

There has been a rich body of literature available on perception, i.e. how sensory information are interpreted in order to represent and understand the environment (see for a review, (Schacter et al. 2011). It is widely acknowledged that perception is not just a passive registration of the sensory input, but it involves an active reconstruction procedure involving learning, memory, expectation, and attention (Bernstein, 2013). Jerome Bruner breaks down the process of perception into three steps (Bruner 1973):

1. Encountering an unfamiliar target/space/environment, we are open to different informational cues and want to learn more about the target.
2. One tries to collect more information about the target/space/environment. Gradually, looking for some familiar cues to help him/her categorize the target or perceive the environment.
3. The cues become less open and selective. We are looking for those cues which affirm his/her categorization of the target. We also actively ignore and even distort cues that violate our initial perceptions. Our perception becomes more selective and we finally paint a consistent picture of the target or perceive an environment.

Extrapolating and interfacing Bruner’s process to perception of environments, a question surfaces: How does the brain react while navigating in an unconventional virtual environment, which possesses none of the qualities of the conventional physical world and which, the brain cannot find any cues to correlate with previous knowledge of space? This question is addressed in the current study.

**Fig. 1:**
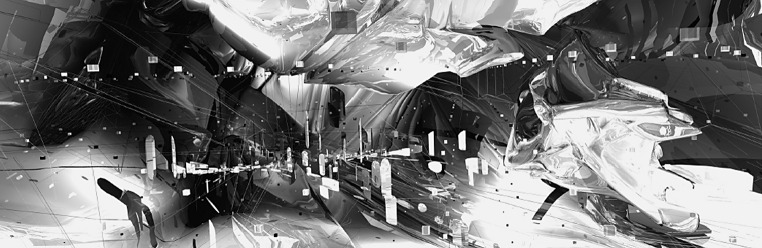
Sample abstract environment. Courtesy of Marcos Novak-V4D_Visio4D

Abstraction is the process of taking away or removing characteristics from something in order to reduce it to a set of essential characteristics. In other words, it is an act of considering something as a general quality or characteristic, apart from concrete realities, specific objects, or actual instances (Langer 1953). The ‘Object’, which remains, after abstraction in Abstract artworks is a representation of the original, with unwanted detail omitted. In his classical book “*Visual Thinking*” Rudolph Arnheim explains “Abstract art” as a visual language of form, color and line to create a composition which may exist with a degree of independence from visual references in the world (Arnheim 1969). Narrowing down the concept of abstraction to architectural space, the definition can be modified as follows: Abstract architectural environments are those, which use a visual language of form, color and line to create a composition which may exist with a degree of independence from visual references in the physical world. In the current research context, “degree of independence” is considered as “not complying with physical rules, e.g. lack of gravity, infinite depth, continuous change and whatever that is not perceivable in the physical world. Abstract environments are subjective. They may be interpreted and perceived in more than one way and lack one unique perception. Seeing all abstract environments typically lack scale and no clear measure to understand the environment clearly (Fig. 1).

In this research we experimented with three different designed environments: abstract, semi-designed and fully designed. Healthy human adults virtually navigated in these three types of design environments while their brain responses were recorded. We predicted distinct brain responses in higher order brain areas, typically associated with planning and executive functions, would be differentially engaged with navigating in these three designed environments.

## Materials and methods

### Participants

Twenty-one healthy human adults (aged 18–39 years, mean 23 years, 17 female) with normal hearing (self-reported) and normal or corrected-to-normal vision participated in the experiment. All participants were recruited from the campus at Goldsmiths, University of London. None of the participants had any architectural background, however some of them were from the department of Design. All participants were in good mental health, and had no past history of neurological illness. Data from one participant was discarded due to poor quality of the EEG signals. All participants provided written informed consent before starting the experiment. The study was approved by the local Ethics Committee of the Department of Psychology at Goldsmiths and conducted in accordance with the Declaration of Helsinki.

### Stimuli

**Fig. 2:**
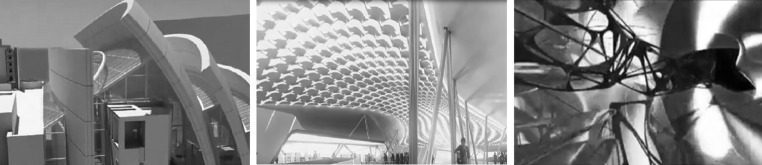
Samples of three different types of design environment: fully designed (*left panel*), semi-designed (*middle*), and abstract design (*right*). Here only a snapshot of individual design is shown and in the actual experiment we presented a short video (1 min long) in each category

The stimuli consisted of fifteen videos of architectural environments, simulating three design categories; fully designed, semi-designed and abstract design. Figure 2 shows an individual sample of the three categories. There were five videos for each category and the duration of each video was 1 min.

The architectural simulations have been created by different 3D software, e.g. 3Ds Max, Revit, Rhino and Grasshopper. The differences in the 3D interfaces were not the intention of the authors as long as the content conforms to the categories. Having the same resolution, all videos were transformed to the VGA format (640 × 480 pixels). Choosing the videos and categorization happened subjectively by the authors.

### Experimental procedure

**Fig. 3:**
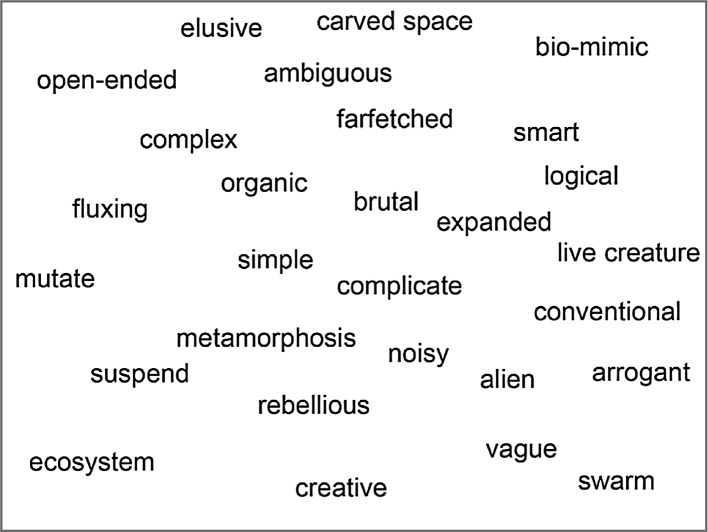
List of buzz-words. At the end of each video, participants were instructed to choose five words from this list that they would consider best fit to the environment

Participants were seated in front of a computer in a dimly lit room. The experimenter placed an EEG cap on their head to monitor their brain’s electrical activity during the experiment. The participants were informed that they would be presented with different design videos and were instructed to look at the video carefully. The order of the video was randomized across participants. At the end of each video, the participants were instructed to rate, on a 7-point Likert scale, three aspects of the design environment as follows: (1) the ease of navigation within the environment, (2) the creativity of the design, and (3) their personal liking of the environment. Further, participants were asked to choose around five words from the list of buzzwords (Fig. 3), which would best describe the qualities and characteristics of the environment of the video shown immediately before. They were also allowed to add their own words if they could not find any appropriate word from the presented list to describe the environment of the video. The participants were presented with a practice video at the beginning to get them familiarized with the experimental procedure.

### EEG recordings

The EEG signals were recorded by placing Ag–AgCl electrodes on 32 scalp locations according to the extended International 10–20 electrode placement system (Jasper 1958). The electrode AFz was used as ground. The EEG signals were amplified (Synamps Amplifiers, Neuroscan Inc.), filtered (dc to 100 Hz), and sampled at 500 Hz. EEG data were re-referenced to the arithmetic mean of the left and right earlobe electrodes (Essl and Rappelsberger 1998). The vertical and horizontal electro-oculograms were recorded in bipolar fashion to monitor eye blinks and eye movements. All electrode impedances were kept below 5 KΩ.

### EEG pre-processing

Prior to analysis, EEG signals were first visually inspected for identification of large artifacts (e.g., excessive muscular artifacts). Next we applied Independent Component Analysis (ICA), a blind source separation method (Jung et al. 2001; Lee et al. 1999; Naganawa et al. 2005), to transform EEG signals into maximally statistical independent components (ICs). We removed those ICs that are primarily related to vertical eye-blinks and horizontal saccades and re-transformed back to the EEG signal space. Afterwards, epochs with the duration of 1 min for viewing individual design environment were extracted, and finally subdivided into non-overlapping ten segments each with 10 s long. All preprocessing were done by the Matlab Toolbox EEGLAB (Delorme and Makeig 2004).

### EEG source localization

The standard low-resolution brain electromagnetic tomography (sLORETA) was used to compute the cortical three-dimensional distribution of current density. It computes the inverse solution by using a realistic head model based on the MNI152 template (Mazziotta et al. 2001), with the three-dimensional solution space restricted to cortical gray matter, as determined by the probabilistic Talairach atlas (Lancaster et al. 2000). A spatial resolution of 5 mm was used, producing 6239 voxels. Thus the sLORETA image represented the standardized electrical activity at each voxel in neuro anatomic Montreal Neurological institute (MNI) space as the exact magnitude of the estimated current density (Musso et al. 2010).

The sLORETA software package (Pascual-Marqui 2002) was used to compute average cross-spectral matrices for 8 standard EEG frequency bands: delta (1.5–6 Hz), theta (6.5–8 Hz), alpha1 (8.5–10 Hz), alpha2 (8.5–10 Hz), beta1 (12.5–18 Hz), beta2 (18.5–21 Hz), and beta3 (21.5–30 Hz), providing a single cross-spectral matrix for each participant, frequency band and design condition, from which we computed the current source density (CSD). Subsequently, CSD values were log-transformed. Next, we performed three pairwise statistical comparisons to explore the differences in brain activation patterns separately for fully designed vs abstract, abstract vs semi designed, and semi designed versus fully designed. For each comparison, we performed non-parametric statistical analysis, which was based on estimating the empirical probability distribution of the maximum *t* statistic under the null hypothesis of no differences, via 5000 randomization, and corrected for multiple comparisons of all 6239 voxels (see Nichols and Holmes 2002), for details on this statistical permutation procedure).

## Results

### Behavioural responses

**Fig. 4:**
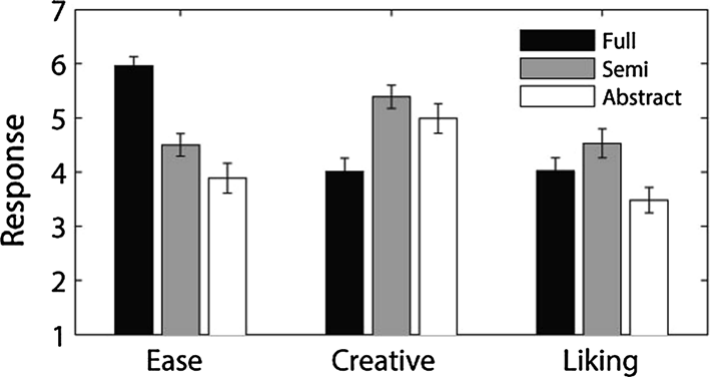
Mean responses on the ease (of navigation), creativity and liking of the three types of design environments, full, semi and abstract

First, we analysed the three behavioural ratings (on the ease of navigation, creativity and liking) provided by the participants at the end of each video. Figure 4 shows the mean responses of these three ratings three types of design environments. A 3 × 3 within-subjects factorial ANOVA was performed with the following factors, *design* (3 levels: full, semi, and abstract) and *response* (3 levels: ease of navigation, creativity and liking). There were main effects of *design* (*F*(2, 38) = 5.40, *p* = .01) and response (*F*(2, 38) = 10.05, *p* = .002) and an interaction effect between *design* and *response* (*F*(4, 76) = 24.18, *p* < .001). Follow up tests suggests that fully designed environments, as expected, were rated easier to navigate than both semi (*F*(1, 19) = 54.41, *p* < .001) and abstract (*F*(1, 19) = 46.98, *p* < .001) design environments, whereas the semi designed environments were judged as slightly more easier to navigate than the abstract (*F*(1, 19) = 6.66, *p* = .02) ones. However, fully designed environments were judged as less creative than the other two ones (*p* < .01), but the differences in creative rating between the semi and abstract design environments were not statistically significant (*F*(1, 19) = 3.44, *p* = .08). The semi design environments were subjectively most liked by our participants followed by fully design and abstract design environments.

**Fig. 5:**
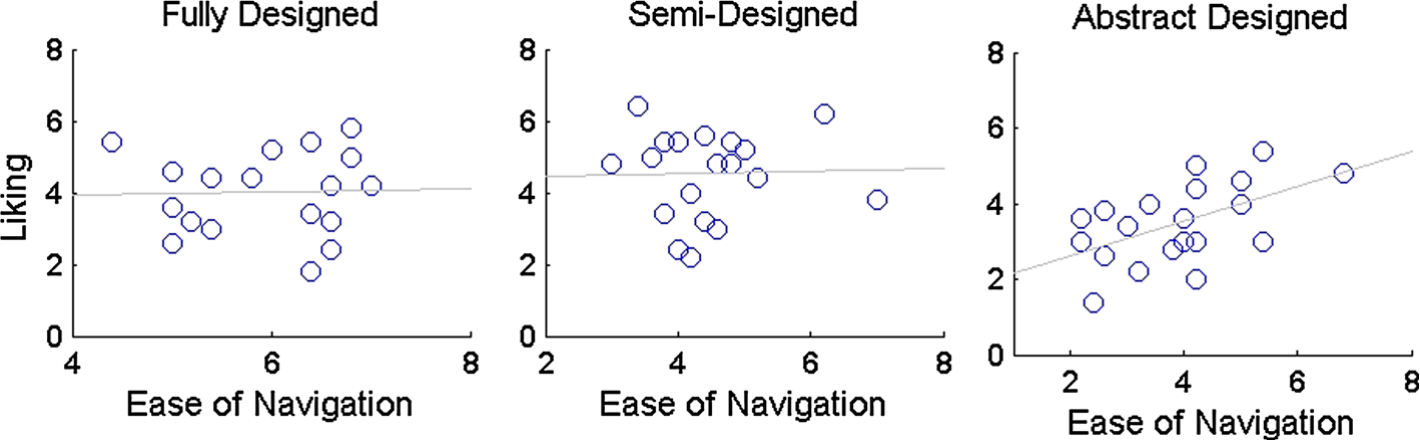
Scatter plots of ease of navigation versus liking for three design environments. *Note* only the abstract designed environment shows a clear relationship (*r* = .54)

Next we studied the interrelationships between these three responses by performing pairwise Pearson’s product-moment correlations and the correlation values are listed in the Table 1. We found that the ease of navigation within an environment was not related with the creativity judgment (*r* ≈ 0). However, if a design environment was judged to be more creative it was also more liked and vice versa, and this relationship was slightly stronger in the full and semi design environments than the abstract ones. The most surprising observation was that the ease of navigation was not related to the liking judgment for both full and semi design environments, yet a strong relationship was found for abstract design (Fig. 5).

**Table 1:** Correlation values between three behavioral ratings in three design environments

	Fully designed	Semi-designed	Abstract designed
Ease × creative	−.03	−.04	.05
Ease × liking	.03	.03	.54
Liking × creative	.74	.75	.62

### Buzzwords responses

**Fig. 6:**
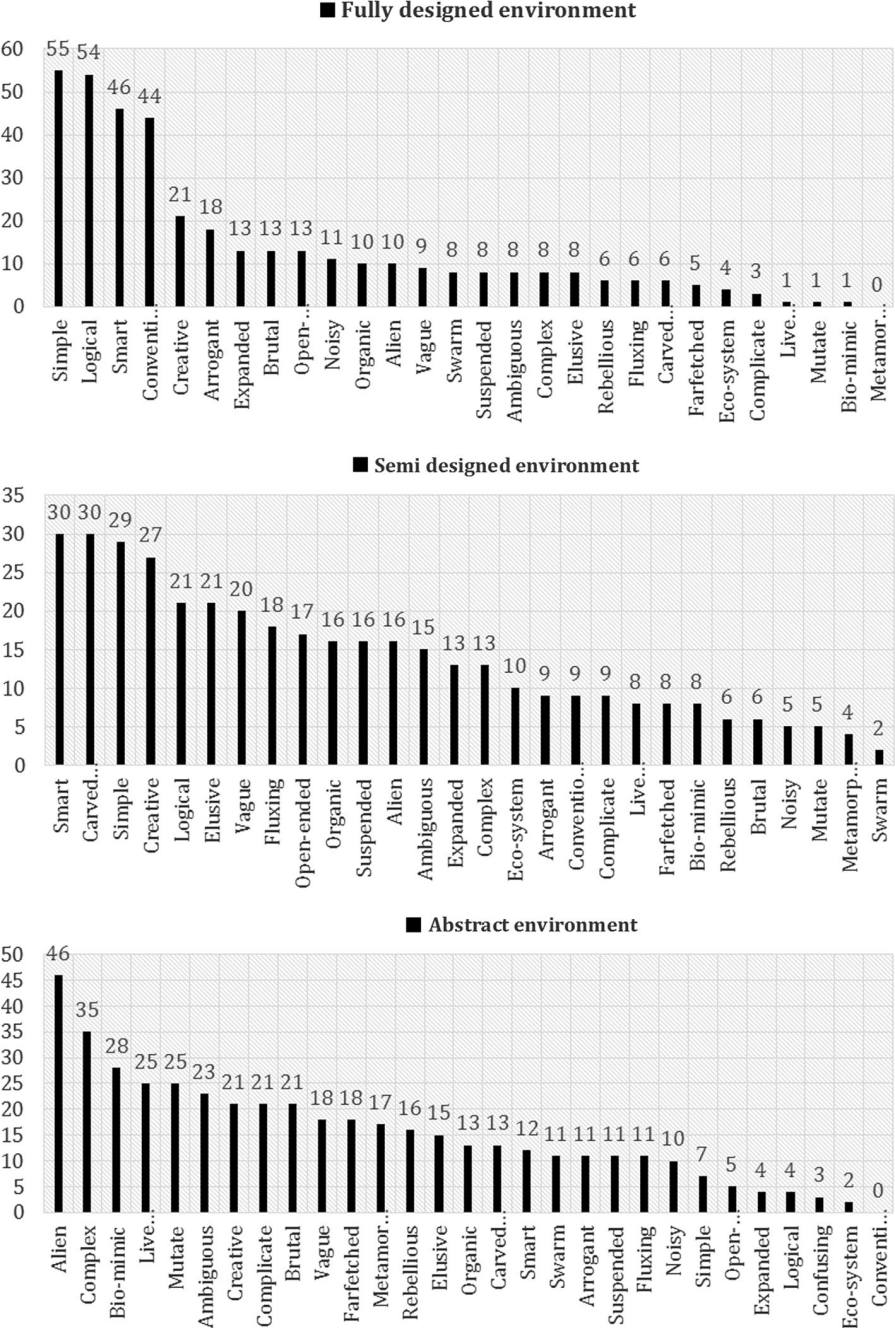
Distribution of buzzwords selected to represent three types of design environments: fully designed (*top panel*), semi designed (*middle panel*), and abstract designed (*bottom panel*)

Next we looked at the selection of buzzwords for the three design environments (Fig. 6). The number of buzzwords used for each category describes the characteristics of that environment. Participants chose “simple, logical, smart and conventional and less metamorphosis, mutate and bio-mimic” traits for a fully designed environment. These environments were not open to different interpretations. Further, participants were quite consistent with their selections in representing the fully designed environment (as reflected by a sharp fall after four buzzwords). For the semi designed environment, participants frequently chose “smart, carved space, simple and creative and less swarm, metamorphosis and mutate.” The abstract design environment was associated with buzzwords such as “alien, complex, bio-mimetic, lively creature mutation, and ambiguous” and much less frequently other buzzwords such as “conventional and logical”. Interestingly, among the three design environments, semi design one was associated with more varied response across participants (as reflected by a stronger trend towards a uniform distribution). Altogether, these observations fit well with the distinction between abstract, fully designed and semi-designed environments that were targeted in our experimental design. The data also showed that the abstract environments require more interpretation (rather than receiving more details, dimensions, scale, etc. in a fully and semi designed environments) and associated with dynamical attributes that are further biologically rooted.

### EEG power analysis

A three-way repeated-measures ANOVA with the following factors, electrode location (32 channels), condition (abstract, semi-designed, fully designed), and frequency band (delta, theta, alpha, beta) as within-subjects factors on average EEG power showed significant main effects of location (F(5.09, 96.64) = 11.33, *p* < .001), frequency (F(1.14, 21.58) = 444.76, *p* < .001), and a location × frequency interaction (F(5.67, 107.60) = 17.01, *p* < .001).

Analysis of variance over all 7 frequency bands (delta, theta, alpha1, alpha2, beta1, beta2, and beta3) showed a main effect of condition on absolute global power, *F*(2, 57) = 3.22, *p* = .047. Post-hoc testing showed that this effect was strongest for the beta2 frequency band, *F*(2, 57) = 8.27, *p* < .001.

### EEG source localization

**Fig. 7:**
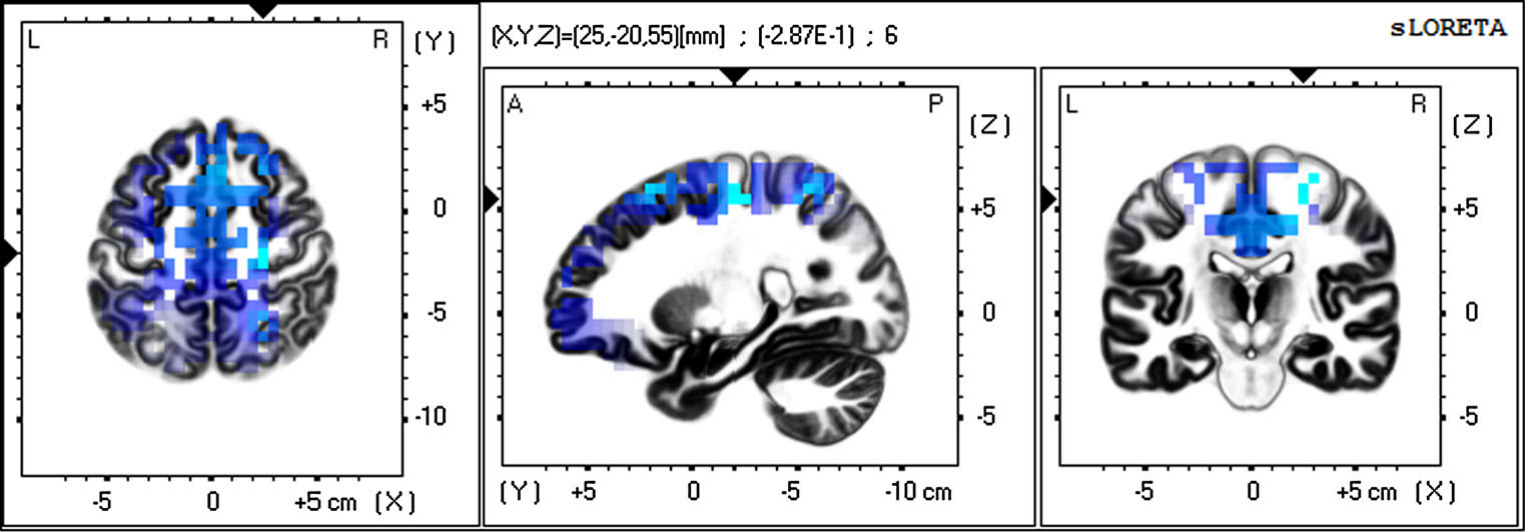
Activation of the precentral gyrus in the fully designed condition versus the abstract condition

Source reconstruction at the whole brain level was performed using the sLORETA method, and statistical comparisons were performed pair-wise between any two conditions. For the fully designed vs abstract designed comparison, we detected a decrease in the beta2 activity primarily in the precentral gyrus (Brodmann area 4), followed by activation from the anterior cingulate (BA 24). Beta3 activation showed a somewhat smaller difference between the two environments (*t* = −.264, *p* = .02), and was located more anterior, potentially originating in the superior prefrontal gyrus (BA 6). These areas showed more activity in the *fully designed* condition than in the *abstract* condition. We did not find significant results in any other frequency band (Fig. 7).

- Abstract versus semi-designed

Similar to the fully designed versus abstract environment comparison, the biggest difference in activation was found in the precentral gyrus (BA 6), but this time in both beta2 as well as beta3 frequency bands (*t* = −.466, *p* = .001).

**Fig. 8:**
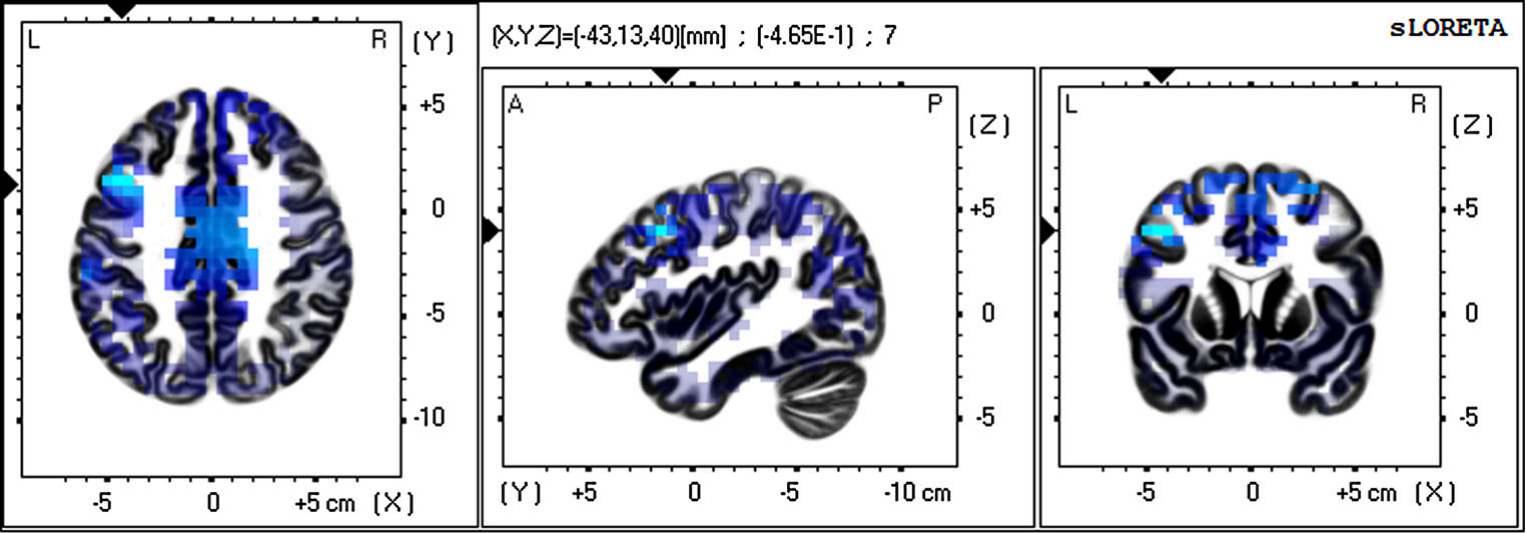
Activation of the dorsolateral prefrontal cortex in the abstract condition

Also, the dorsolateral prefrontal cortex (BA 9) showed more beta3 activity in the abstract condition compared to the semi-abstract condition (*t* = −.465, *p* = .001, see Fig. 8). We did not find significant results in any other frequency band.

- Semi versus full

No robust significant differences were observed between the semi-abstract and full conditions (all *p*s > .097).

The results of different comparisons are summarized in the Table 2.

**Table 2:** Summary results of three comparisons based on sLORETA findings

	Delta	Theta	Alpha1	Alpha2	Beta1	Beta2	Beta3
Fully designed versus abstract	–	–	–	–	–	Precentral gyrus (BA4), anterior cingulate cortex	Superior prefrontal gyrus (BA6)
Semi designed versus abstract	–	–	–	–	–	Precentral gyrus (BA6)	Precentral gyrus (BA6), dorsolateral prefrontal cortex (BA9)
Fully designed versus semi designed	–	–	–	–	–	–	–

## Discussion

Architecture is a multi-faceted discipline, which involves the act of visualizing, designing (divergent thinking) and problem solving (convergent thinking) as an iterative process. It is important for a designed to understand how our brains navigate in a designed environment, as the understanding is inextricably linked to the whole design procedure. By navigating in three different virtual environments, the perception of abstract virtual environment is different from fully designed or semi designed environment. Applying abstract design in early stages of design procedure may help the brain to think as divergent a possible and ease the visualization and form-finding.

Across the studied standard seven EEG frequency bands, the most robust differences across all three comparisons were found in the beta2 and beta3 frequency bands. Synchronized neuronal oscillations at the broad beta frequency band (13–30 Hz), covering both the beta2 and beta3 bands, are usually prominent in the human motor system, including somatosensory cortex, basal ganglia and the cerebellar network (Jenkinson and Brown 2011). Therefore, beta oscillations are often linked to diverse range of sensorimotor functions such as planning, preparation and execution of movements (Pfurtscheller et al. 1996; Salmelin et al. 1995); (Pavlidou et al. 2014). Further, sensorimotor beta oscillations are also involved with observation and imagination of biological movements (Muthukumaraswamy and Johnson 2004; Schnitzler et al. 1997) These evidence have led to the suggestion that oscillatory beta activity over the sensorimotor network represents a matching mechanism to internally stored mental representations of actions, and subsequently provides the substrates for the functional integration of visual and sensorimotor brain regions (Pavlidou et al. 2014). Altogether this also confirms the appropriateness of the designed environments presented in our study.

We also found consistent differences in brain activation patterns in the motor network involving precentral gyrus associated with perceiving abstract design environments. This is in line with the body of literature demonstrating the role of sensorimotor areas in aesthetical appreciations, especially of abstract art (Freedberg and Gallese 2007; Hagerhall et al. 2008; Jacobsen et al. 2006; Umilta et al. 2012). We could not speculate on the artistic value of our abstract design environment, but it is likely that the total unfamiliarity of the presented environment might have led the observer, i.e. our participants, to consider more similar to an abstract art form. This further substantiates the notion of embodied cognition in the context of viewing design environments. Unlike previous studies demonstrating the role of sensorimotor network in observation and imagery of various actions (Muthukumaraswamy and Johnson 2004; Salmelin et al. 1995; Schnitzler et al. 1997), our results show that viewing different types of design environments with varying degree of abstractness would differentially impact on viewer’s cortical motor system. Do note though that we do not claim that such motor activation is causally related to the aesthetic experience of the viewer, instead we suggest that this spontaneously evoked cortical motor activation reflects some sort of embodied simulation of the presented environment (Gallese 2005; Gallese and Sinigaglia 2011).

In addition to the cortical motor network, we observed differential activations in other brain area, primarily in the prefrontal cortex, and this includes anterior cingulate cortex (ACC), dorsolateral prefrontal cortex (dLPFC) and superior prefrontal gyrus.

Activation of the anterior cingulate cortex (ACC) while navigating fully designed vs abstract designed environments may suggest an increased involvement of higher level cognitive functions such as attention (Weissman et al. 2005), error detection and conflict monitoring (Bush et al. 2000). Further, activation of dLPFC while navigating in an abstract environment could potentially reflect conflict-induced behavioral adjustment (Mansouri et al. already found connections between them in their research Mansouri et al. 2007). Since characteristics of the abstract environment are totally different from the familiar fully- or semi-designed environments, conflicts and rule violations would be the norm while viewing an abstract environment, yet it is also crucial to resolve these conflicts in a dynamic and adaptive fashion in order to ensure an appropriate mental simulation of the abstract environment.

There are two principal limitations of the current study. First, the selection of the three types of design environments could be considered a bit arbitrary. Although we have carefully tried to choose and categorize the three environments, the selection process happened subjectively as there is no known objective way to categorize the environments in the desired category. Further, the concept of abstractness may be on a continuum yet we considered only three snapshots on this continuous scale of abstractness. Secondly, it is not clear whether the reported differences in large scale brain activity while navigating abstract virtual environment is any way related to the aesthetics and/or creativity of the presented design.

## Conclusion

Architecture is a multi-faceted discipline and the design process is always seen as an iteration cycle between design and problem solving. The functioning of the brain is completely different while doing these two tasks and therefore it is important for an architect to know the mechanisms of his/her brain in order to find efficient and more effective combinations between these two tasks. The brain function is different while perceiving an abstract environment as compared to the perception of a fully designed or semi-designed environment. Navigating abstract virtual environment requires more precentral efforts comparing with fully or semi-designed environment. Therefore, starting the early stages of design with an abstract environment with a degree of freedom from all physical rules, restrictions and confinements may help one to think as divergent as possible and thus be more creative during the idea generation phase of architectural design.
